# Expression of Resistance in *Amaranthus* spp. (Caryophyllales: Amaranthaceae): Effects of Selected Accessions on the Behaviour and Biology of the Amaranth Leaf-Webber, *Spoladea recurvalis* (Lepidoptera: Crambidae)

**DOI:** 10.3390/insects9020062

**Published:** 2018-06-08

**Authors:** Stephen T. O. Othim, Srinivasan Ramasamy, Ruth Kahuthia-Gathu, Thomas Dubois, Sunday Ekesi, Komi K. M. Fiaboe

**Affiliations:** 1 Kenyatta University, School of Agriculture and Enterprise Development, P.O. Box 43844-00100, Nairobi, Kenya; somburo@icipe.org (S.T.O.O.); rkahuthia@gmail.com (R.K.-G.); 2 International Centre of Insect Physiology and Ecology (ICIPE), Plant Health Unit, P.O. Box 30772-00100, Nairobi, Kenya; sekesi@icipe.org; 3 World Vegetable Center, P.O. Box 42, Shanhua, Tainan 74199, Taiwan; srini.ramasamy@worldveg.org; 4 World Vegetable Center, Eastern and Southern Africa, P.O. Box 10, Duluti, Arusha, Tanzania; thomas.dubois@worldveg.org

**Keywords:** antibiosis, antixenosis, IPM, longevity, mortality

## Abstract

*Spoladea recurvalis* F. is a major pest moth of amaranth (*Amaranthus* spp.) flowers worldwide, with a potential of causing complete foliage loss under severe outbreaks. Chemical insecticides are uneconomical for resource-poor farmers and pose health and environmental risks. Host plant resistance (HPR) to insects is an effective, economical and environmentally friendly alternative that is poorly understood and largely unexploited among traditional leafy vegetables. A total of 35 amaranth accessions were evaluated for the expression of their antixenotic and antibiotic traits against *S. recurvalis*, focusing on their effects on the biology of the pest in comparison with a susceptible accession. The accession VI036227 was found to be highly resistant against the pest, exhibiting exemplary antibiosis by causing 100% larval mortality within the first 36 h, despite not being deterrent for oviposition. The accessions VI048076, VI056563 and VI047555-B demonstrated moderate resistance against the pest for specific parameters including low oviposition, moderate early stage larval mortality and reduced adult longevity. Total mortality and weight gain in these three accessions were, however, not significantly different from the susceptible control. Higher numbers of eggs were laid in no-choice compared to choice situations. The implications of these findings in the management of *S. recurvalis* on amaranths are discussed.

## 1. Introduction

Amaranth plants, in the genus *Amaranthus* L. and family Amaranthaceae, are traditional leafy vegetables (TLVs) that are largely consumed in several countries around the world, not only as vegetables (leaves) but also as grains [1,2]. Amaranth foliage has gained widespread acceptance and popularity as a dietary constituent in Africa and Asia [3,4], mainly due to increased awareness of its nutritive and medicinal value as it is rich in vitamins A, B and C, calcium, iron, potassium, ascorbic acid and also provides an alternative source of vegetable protein [1,3,5,6,7,8]. The grains are equally nutritious and are largely used in feeding children and the elderly to boost their immunity by supplying much-needed micronutrients and as a major source of relief for the lactose-intolerant [1,9]. In addition to nutrition, amaranth also possesses qualities such as earliness to mature, palatability and adaptability to a broad range of climatic conditions, including temperature, moisture and water stress, assisted by its C_4_ photosynthetic pathway [1]. Most amaranth species have also been shown to tolerate moderate salinity levels [10].

Arthropod pests and diseases present a major challenge to optimum productivity of amaranths in several regions of the world including Africa [11,12,13,14], Asia [15] and the Americas [7,16,17]. Currently, more than 250 species of insect pests have been reported to feed on amaranth worldwide, with a majority (50%) falling in the category of leaf chewers/defoliators [11,16,17,18].

In Africa and Asia, the Hawaiian beet webworm/amaranth leaf-webber, *Spoladea recurvalis* (Fabricius, 1775) (Lepidoptera: Crambidae), has often been reported to be a major pest in amaranth fields, with a potential of causing complete defoliation of foliage under severe outbreaks [11,19,20,21,22]. The eggs of *S. recurvalis* are normally laid on the lower surface of leaves adjacent to leaf veins, from where they hatch and begin to feed on the crop [23]. Young larvae of *S. recurvalis* feed beneath the leaves only on the epidermis, skeletonising the tissues and occasionally spinning light webs in which they rest [4]. After the second instar, they skeletonise the foliage, leaving only the main veins intact, roll the leaves into distinctive leaf shelters, form webbing on leaves and leave frass on the leaves [4,12,24]. The webbing and rolling of the leaves severely diminishes the photosynthetic capacity and often leads to the death of the plant.

In several countries, management of this pest has been through the use of synthetic insecticides [11,16,25]. However, insecticide application to control vegetable arthropod pests is not economical under subsistence farming, being largely beyond the means of resource-poor farmers; it also induces resistance in pests and is often inefficient [26,27,28]. Amaranth, being a short season crop, poses the inevitable temptation of farmers using the chemicals indiscriminately and non-judiciously, leading to environmental pollution and elimination of natural enemies [28]. Moreover, health concerns due to residue levels in vegetables have often been raised concerning these pesticides, thus the need for development of effective, safe and sustainable Integrated Pest Management (IPM) approaches [25,26,28]. The moth attractant phenylacetaldehyde (PAA), which has been demonstrated to be effective in capturing *S. recurvalis* in Hawaii, was not effective in capturing the East African populations [21,22,29].

In light of these developments in the management of *S. recurvalis*, host plant resistance (HPR), though greatly unexploited among TLVs, assumes a pivotal role in the management of pests of amaranth, particularly *S. recurvalis*. Studies have shown that different varieties, accessions or lines of amaranth exhibit different levels of resistance in terms of herbivory and infestation by pests including *S. recurvalis* in open field conditions [7,30]. The mechanisms of such resistance in amaranths are, however, still unknown. This study therefore sought to unravel the mechanisms of resistance with a special focus on expression of antixenosis and antibiosis against *S. recurvalis* among selected resistant accessions of amaranth.

## 2. Materials and Methods 

### 2.1. Amaranth Accessions

Open field experiments were conducted in 2016 and 2017 to screen 31 amaranth accessions obtained from the World Vegetable Center (WorldVeg) gene bank in Taiwan and four improved lines from WorldVeg Eastern and Southern Africa (ESA) for resistance against leaf-webbers [30]. In this study, we conducted a further screening for expression of resistance in a screenhouse on all 35 test accessions and lines (hereafter both called accessions) and one susceptible accession in choice assays. Morphological characteristics of the amaranth accessions tested are presented in Table 1. From both the open field assay previously conducted by Othim et al. [30] and the screenhouse assays conducted in this study, eight amaranth accessions exhibiting pest resistance and the susceptible accession were selected on the basis of damage incidence and severity, pest incidence and abundance and oviposition preference for in-depth assessment of their effects on selected biological traits of the pest. The susceptible accession was considered as such because it had the most severe damage and the highest pest incidence and abundance. The selected accessions were grown in the screenhouse at the WorldVeg facility in Arusha. The seeds were sown in plastic trays containing a substrate of soil and manure in the ratio 4:1. Two to three weeks after germination, the seedlings were transplanted into plastic pots of 10 cm diameter (1000 cm^3^) and maintained with regular watering for use in the experiments. For pest colony maintenance, *Amaranthus dubius* Mart. ex Thell. (Ex-zan), obtained from WorldVeg’s ESA genebank in Arusha, Tanzania, was used.

### 2.2. Amaranth Leaf-Webber (Spoladea recurvalis F.) Colony

A colony of the amaranth leaf-webber was established and maintained in the entomology laboratory at WorldVeg, Arusha on *A. dubius* for five generations prior to their experimental use. The adults and larvae of *S. recurvalis* were originally collected from amaranth fields within WorldVeg (3.38° S, 36.8° E) in November and December 2015. Adult moths were placed in transparent Perspex cages (40 × 40 × 45 cm) with a sliding door and a netting material at the back and on the sides for ventilation. The moths were fed on 10% honey solution soaked in cotton wool and provided with potted amaranth plants for oviposition. The plants were replaced every 24 h and placed in separate holding cages (50 × 50 × 60 cm) made from transparent Perspex material with netting at the back and on the sides for the eggs to hatch. Newly hatched larvae were left to feed on the live plants for three to four days and then transferred into plastic containers (15 × 7 × 5 cm) lined with paper towel and fine netting material on the lid for ventilation. Fresh amaranth leaves were supplied to the larvae daily for food until pupation. The pupae were incubated under similar conditions in the plastic containers until adult emergence. The laboratory conditions were maintained at 25 ± 2 °C, 50–70% RH and photoperiod of 12:12 h (light:dark).

### 2.3. Choice Bioassay

Choice assays were conducted in two sets since all the 36 accessions could not fit in a single cage. The experiment was conducted within a screen-house in a split plot design replicated six times. In the first set (set-up A), 17 test accessions and the susceptible accession were exposed to 25 mated female adults of *S. recurvalis* in a glass cage measuring 150 × 100 × 120 cm. In the second set (set-up B), established three weeks later, the 18 remaining test accessions and the susceptible accession were exposed to 25 mated female adults of *S. recurvalis* in the same glass cages used for the first set. One potted plant of each accession (seven weeks old) was randomly placed in each cage and left for 48 h for the moths to lay eggs. The plants were watered after 24 h and the moths were provided with 10% honey solution on cotton plugs to feed. The plants were then removed from the cages and the leaves of each plant thoroughly inspected for the presence of eggs, which were counted with the aid of a dissecting microscope and recorded.

### 2.4. No-Choice Bioassay

The eight most resistant accessions identified from the field experiments conducted by Othim et al. [30] and the choice experiments above were assessed individually in a no-choice experiment in comparison with the susceptible accession. One potted amaranth plant of each selected accession at six to seven weeks of age was exposed to two mated female moths of *S. recurvalis* from the stock culture for 48 h in the transparent Perspex cages. During the exposure period, *S. recurvalis* adults were fed on 10% honey solution soaked in cotton wool and the plants were watered adequately. After 48 h of exposure, the plants were removed from the cages and the leaves were inspected for the presence of eggs under a dissecting microscope. The number of eggs on each amaranth accession was recorded. This experiment was replicated six times with each accession.

### 2.5. Effect of Accession on Weight Gain of Amaranth Leaf-Webber

One or two leaves (based on leaf size) from each of the eight selected amaranth accessions and the susceptible accession were exposed to one larva of *S. recurvalis* for 48 h in a petri dish lined with moistened filter paper. The leaves were obtained from amaranth accessions grown and maintained in the screen-house at six to seven weeks. The larvae were obtained from the laboratory stock culture and exposed at 3–5 days old to each accession. Prior to their exposure in the experiment, the larvae were deprived of food for 12 h. The weight of each larva was measured before and after 48 h of exposure to the leaves using a digital scale (Mettler AE200 analytical balance, Columbus, OH, USA). This experiment was replicated 12 times with each amaranth accession. The weight gain and percentage weight gain by *S. recurvalis* larvae on each accession were then calculated.

### 2.6. Effects of Selected Amaranth Accessions on the Development of Amaranth Leaf-Webber and Adult Longevity

The nine accessions tested in no-choice experiment were evaluated for their effect on larval development. Five neonate larvae of *S. recurvalis* were placed in a plastic Petri dish (8 cm diameter) lined with filter paper to absorb excess moisture. These were supplied daily with fresh leaves of the selected accessions until all larvae had pupated or died. The pupae were then incubated under the same conditions until adult emergence. The emerged adults were placed in Perspex cages and fed on 10% honey solution until they died. The assay was replicated 10 times with each selected amaranth accession. The data on larval, pupal and total developmental times, larval and pupal mortalities and adult longevity were collected for each accession. Early stage larval mortality was recorded as mortality within the first 36 h (when the larvae are not causing considerable damage) of exposure.

### 2.7. Data Analysis

One-way analysis of variance (ANOVA) was used to compare morphological characteristics of amaranth accessions including number of branches per plant, plant height, leaf length and width and petiole length using GENSTAT version 19.1. All other statistical analyses were performed using R-Software version 3.4.0 (R Core Team, 2017). The count data were analysed using generalized linear model (GLM) with log_10_-link and Poisson distribution error to compare the factors: number of eggs oviposited by female *S. recurvalis* moths from both choice and no-choice assays, number of days taken for larval, pupal and total development by *S. recurvalis* and adult longevity on various accessions. The effect of a factor for a GLM is reflected in the deviance (likelihood ratio test statistic) that has an appropriate chi-square distribution; hence the chi-square values are presented as test statistics. The “Relative Risk/Risk Ratio” (RR), which is a ratio of the probability of having the pest lay an egg on the test amaranth accession relative to the susceptible accession, was calculated as an exponent of the coefficients obtained from the Poisson regressions. The number of eggs obtained in the choice and no-choice assays was compared using a chi-square goodness-of-fit test. The percentage weight gain and weight gain (mg) by larvae of *S. recurvalis*, larval and pupal mortalities, egg viability, fecundity and F_1_ female proportions were analysed using one-way ANOVA. The percentage weight gains by larvae of *S. recurvalis* and larval and pupal mortalities were square-root transformed before ANOVA, and Tukey’s test used to separate means where significant differences occurred. The instantaneous rate of increase (*r_i_*)was calculated according to Stark and Banks [31] using the following equation:

*r_i_*= ln(N_f_/N_o_)/T

where N_f_ is the final number of insects, N_o_ is the initial number of insects, and T is the change in time (number of days the experiment was run). Positive values of *r_i_* indicate a growing population, *r_i_* = 0 indicates a stable population, and negative *r_i_* values indicate a population in decline and headed toward extinction. Spearman’s rank order correlation analysis was conducted to establish the existence of relationships between larval vs. pupal mortalities and larval mortality vs. time taken before mortality in *S. recurvalis*.

## 3. Results

### 3.1. Morphological Characteristics of Amaranth Accessions

Amaranth accessions exhibited different morphological characteristics including leaf coloration, leaf shape, leaf size and growth habit among others. The susceptible accession had significantly broader (*F* = 37.9; df = 35,178; *p* < 0.001) and longer (*F* = 31.1; df =35,178; *p* < 0.001) leaves compared to the resistant accessions (Table 1). The smallest leaf sizes were recorded on accession VI036227 with width of 1.2 ± 0.1 cm and length 3.2 ± 0.7 cm compared to 10.6 ± 0.6 and 19.3 ± 2.2 cm in the susceptible accession. The plant height and petiole lengths also differed significantly among the amaranth accessions. There was no significant difference in the number of branches across all the accessions. Leaf coloration and shape also varied among the accessions with accessions VI046233-A, VI033477 and VI056563 possessing red leaves compared to the green leaves in the susceptible accession (Table 1).

### 3.2. Oviposition by S. recurvalis in Choice Situation

In oviposition choice assays, there were significant differences in the number of eggs oviposited on the tested accessions in set-up A (χ^2^ = 284.03; df = 17, 85; *p* < 0.001) and set-up B (χ^2^ = 1056.40; df = 18, 93; *p* < 0.001) (Table 2). In set-up A, the susceptible accession had significantly higher number of eggs compared to all the other 17 accessions. The fewest eggs were recorded on accessions VI044432 and VI054569, which had a relative risk (RR) of 0.06 compared to the susceptible accession. In set-up B, the accession VI048919 (RR = 1.39) recorded significantly higher number of eggs compared to the susceptible accession while accession VI050609-B (RR = 1.20) did not differ significantly from the susceptible accession in the number of eggs laid by *S. recurvalis* (Table 2). The remaining 16 accessions had significantly lower number of eggs compared to the susceptible accession. The average number of eggs laid by *S. recurvalis* across all the accessions in both set-up A and B was 7.80 ± 0.85 and ranged between 1.50 ± 0.56 in the least preferred accession to 40.20 ± 16.41 in the most preferred accession (Table 2).

### 3.3. Oviposition by S. recurvalis in No-Choice Condition

In the no-choice test, where a total of eight accessions were compared to the susceptible one, *S. recurvalis* laid more eggs on the susceptible accession compared to all the other accessions (χ^2^ = 192.75; df = 7, 37; *p* < 0.001) (Table 3). The accession VI048076 recorded the least number of eggs (18.50 ± 6.63) with a RR = 0.31 compared to the susceptible accession and was also significantly lower than all the other seven accessions. The accessions VI044437-A (RR = 0.49), VI047555-B (RR = 0.49) and RVI00053 (RR = 0.50) also had fewer eggs compared to VI049698 (RR = 0.72). Accession VI036227 (RR = 0.67) had a higher number of eggs compared to accessions VI048076 and VI044437-A, but did not differ significantly from accessions RVI00053, VI047555-B, VI049698 and VI056563 (Table 3). The number of eggs laid by *S. recurvalis* in the no-choice assay was significantly higher than those laid in the choice situation (χ^2^ =1305.10; df = 1; *p* < 0.001).

### 3.4. Weight Gain by Larvae of S. recurvalis after 48 h of Feeding on the Selected Amaranth Accessions

**Figure 1:**
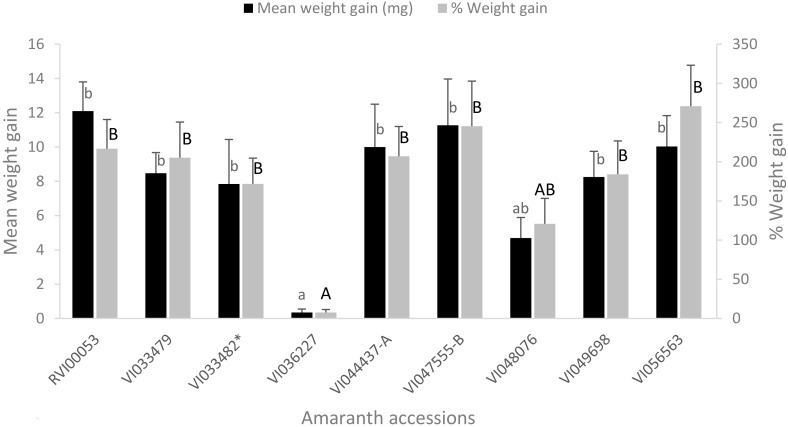
Weight gain (mg) and percentage weight gain (mean ± SE) by larvae of *S. recurvalis* when fed on different amaranth accessions for 48 h. (Mean weight gain (% weight gain) with the same lower (upper) case alphabet is not significantly different at *p* < 0.05, (Tukey’s test)). * Susceptible accession.

The weight gained (mg) by larvae of *S. recurvalis* after feeding on the eight selected amaranth accessions for 48 h differed significantly (*F* = 6.13; df = 8, 99; *p* < 0.001) with accession VI036227 producing the lowest weight gain. Accessions RVI00053, VI033479, VI044437-A, VI047555-B, VI049698 and VI056563 did not differ significantly from the susceptible accession in the weight gain but were significantly higher than accession VI036227 (Figure 1). The average weight gain and percentage weight gain by *S. recurvalis* was 8.11 ± 0.68 mg and 181.0 ± 14.86%, respectively (Figure 1).

### 3.5. Development Time of S. recurvalis on the Selected Amaranth Accessions

The average larval development time was 13.5 ± 0.12 days across the eight selected accessions and ranged between 13.23 ± 0.3 and 14.0 ± 0.56 days on VI049698 and VI056563, respectively. Larval development on accession VI036227 did not advance beyond two days of exposure to the accession and hence larval development on this accession could not be determined. When presented with leaves from accession VI026227, the larvae of *S. recurvalis* gnawed only a small portion of the leaf and in some cases did not even attempt to feed on the leaves. There were no significant differences in the larval (χ^2^ = 1.07; df = 7, 228; *p* = 0.994), pupal (χ^2^ = 3.35; df = 7, 112; *p* = 0.851), and total (χ^2^ = 1.04; df = 7, 112; *p* = 0.994) development times of *S. recurvalis* across the tested accessions (Table 4). The mean pupal development time across all the accessions was 6.36 ± 0.13 days and ranged between 5.86 ± 0.17 and 7.45 ± 0.69 days on VI044437-A and VI033482, respectively. The mean total development time of *S. recurvalis* was 19.09 ± 0.15 days across the tested accessions, ranging between 18.60 ± 0.45 and 20.0 ± 0.80 days.

### 3.6. Mortality Rates and Instantaneous Rate of Increase of S. recurvalis on Selected Amaranth Accessions

**Figure 2:**
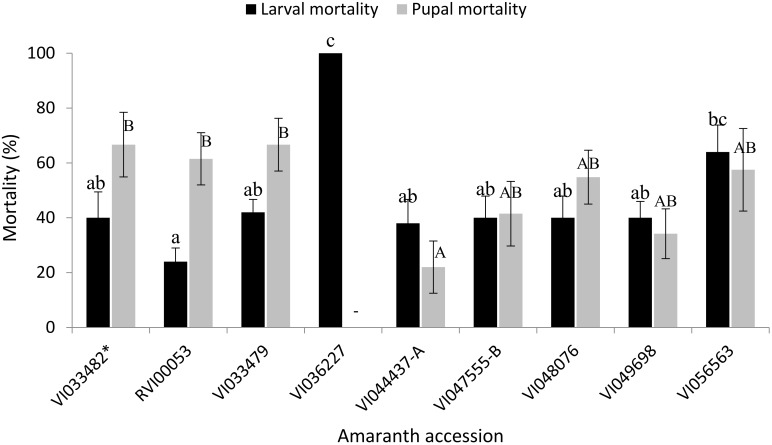
Total larval and pupal mortalities (mean ± SE) of *S. recurvalis* recorded on selected accessions of amaranth. (Means of total larval (pupal) mortality with the same lower (upper) case letters are not significantly different at *p* < 0.05, (Tukey’s test)). * Susceptible accession.

Early stage larval mortality, within the first 36 h, was observed in all the accessions with an overall mean mortality of 25.11 ± 4.56% and ranged between 4.0 ± 0.51 and 100.0 ± 0.0% across the accessions. There were significant differences (*F* = 12.22; df = 8, 81; *p* < 0.001) in early stage larval mortalities that occurred on the different accessions with VI036227 leading to significantly higher mortalities compared to all the other accessions including the susceptible accession (Table 4). The accession VI056563 led to significantly higher early stage mortality compared to RVI00053.The lowest early stage mortalities were recorded on accessions RVI00053, VI033479 and the susceptible check VI033482 (Table 4). The total larval mortalities across the different accessions ranged between 24.0 ± 4.99% and 100 ± 0.0% with an average of 47.56 ± 3.19% (Figure 2). There were significant differences (*F* = 5.91; df = 8, 81; *p* < 0.001) in the larval mortalities when *S. recurvalis* was fed on different amaranth accessions. The accession VI036227 led to 100 ± 0.0% larval mortality, which was significantly higher than larval mortality in all the other accessions except VI056563, which led to 64.00 ± 9.8% larval mortality. The lowest larval mortality was recorded on accession RVI00053, which had a mean mortality of 24.0 ± 4.99% (Figure 2). There was a significant negative linear correlation between the number of days before larval mortality occurred and the rate of larval mortality (*r* = −0.428; *p* < 0.001).

Pupal mortality also differed significantly (*F* = 2.92; df = 7, 68; *p* = 0.01) among the accessions tested. Accession VI044437-A had significantly lower pupal mortalities than RVI00053, VI033479 and the susceptible accession VI033482 (Figure 2). The average pupal mortality was 50.15 ± 4.03%. There was no correlation between larval and pupal mortalities (*r* = 0.064; *p* = 0.58).

**Figure 3:**
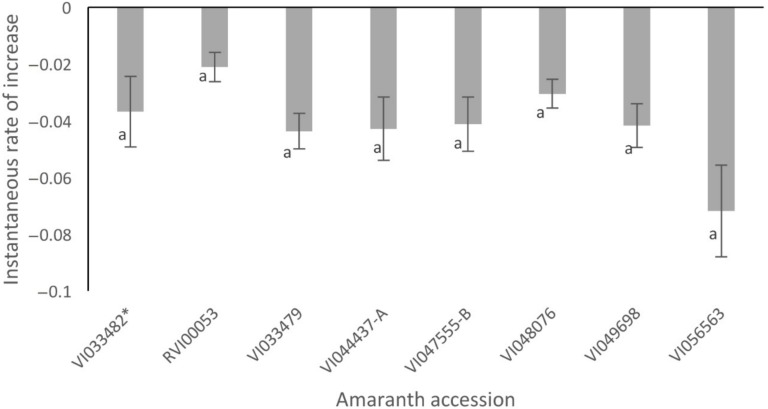
The mean instantaneous rate of population increase (*r_i_*) among larvae of *Spoladea recurvalis* when exposed to different amaranth accessions. Positive values of *r_i_* indicate a growing population, *r_i_* = 0 indicates a stable population, and negative *r_i_* values indicate a population in decline and headed toward extinction (Means with the same letter are not significantly different at *p* < 0.05 (Tukey’s test)). * Susceptible accession.

The instantaneous rate of increase (*r_i_*) when the larvae of *S. recurvalis* were exposed to the different amaranth accessions were negative (Figure 3). The *r_i_* also did not differ significantly among all the tested accessions.

### 3.7. Adult Longevity, Fecundity, Egg Viability and Sex Ratios of S. recurvalis

There were significant differences in the adult longevity among the amaranth accessions (χ^2^ = 92.51; df = 7, 380; *p* < 0.001) with accession VI047555-B producing adults with the shortest longevity (8.7 ± 0.61 days), whereas adults from accession VI048076 had the longest longevity (14.25 ± 0.82 days) (Table 5). Adults obtained from accessions VI056563 and VI048076 had significantly longer longevities compared to adults obtained from the susceptible accession VI033482 and the accessions VI047555-B, VI044437-A and VI033479. Accession VI047555-B also produced adults that had a shorter longevity compared to those from accessions VI044437-A, VI033479, RVI00053, and VI049698 (Table 5). The viability of eggs laid by F_1_ females of *S. recurvalis* that were reared on the different amaranth accessions did not differ significantly (*F* = 0.89; df = 7, 32; *p* = 0.527) (Table 5). Fecundity of the F_1_ females obtained from the various amaranth accessions differed significantly (*F* = 6.07; df = 7,14; *p* = 0.002) with accessions VI049698 and the susceptible accession VI033482 leading to the production of more eggs compared to accessions VI033479, VI044437-A and VI048076 (Table 5). There was no significant difference (*F* = 0.74; df = 7, 25; *p* = 0.638) in the proportions of F_1_ females obtained from the amaranth accessions tested (Table 5).

## 4. Discussion

Amaranth accessions possessed different morphological and physical characteristics such as leaf coloration, shape and sizes. The accession VI036227 had significantly smaller leaves compared to the susceptible accession while accessions VI046233-A, VI033477 and VI056563 had red leaves compared to the green leaves of the susceptible accession. Pest preference for a plant variety has been attributed to the plants’ physical, morphological and chemical features [32,33,34,35]. Physical features like petiole length, breadth of leaf, pigmentation and presence of trichomes have been reported to affect insect pest preference in several crops including amaranths [34,36,37]. Hillier et al. [38] also related pest abundance to the density of plant foliage. In addition, morphological characteristics play an important role in determining farmer and consumer preferences for a variety over others [3]. Therefore, whereas accession VI036227 exhibited high resistance, the tiny leaves it possesses may be a hindrance for its acceptance in certain regions. Similarly, the red coloration of accession VI056563 may inhibit its acceptability certain regions. Further studies are recommended to assess consumer and farmer preferences for selected resistant amaranth accessions.

When offered a choice of hosts for oviposition, *S. recurvalis* exhibited varying levels of preference for the different accessions for oviposition. The accessions VI050609-B and VI048919 did not show antixenosis for oviposition as they had significantly higher number of eggs compared to the susceptible control VI033482. Several accessions (32 out of the 35 tested) exhibited oviposition deterrence, having <50% of eggs recorded in the susceptible control. Accessions VI044432, VI049502 and VI054569 exhibited high levels of antixenosis with <2 eggs compared to 21 eggs in the susceptible control. The choice by an insect to oviposit on a particular plant host and not on the other is usually determined by factors such as plant volatiles, plant anatomy, host nutrition, mobility of immatures, presence of natural enemies and competitors, among others [39]. In the case of *S. recurvalis* on the different accessions of amaranth, it is still premature to predict with certainty which one of these factors played a significant role in antixenosis, but we predict that plant volatiles might be of the most importance. Further research is therefore recommended to determine which of these factors are key in the expression of antixenosis against *S. recurvalis*.

*Spoladea recurvalis* was observed to lay more eggs on the susceptible accession in no choice conditions compared to the selected resistant accessions. This further reiterates the expression of antixenosis for oviposition at varying levels against *S. recurvalis* in these resistant accessions with VI048076 having reduced number of eggs. Significantly fewer eggs were also laid per female in the choice than the no-choice conditions on all the accessions. According to Grovida [24], *S. recurvalis* is largely restricted to plants in the family Amaranthaceae and can be said to be a specialist. In seeking oviposition sites, specialists are usually under pressure to find suitable hosts and prioritize hosts that will offer quality nutrition for their offspring [39,40]. There is, therefore, a likely trade-off by *S. recurvalis* between the number of eggs and time spent by the female in seeking for a suitable host in the choice conditions compared to no-choice situations. In addition, competition between the female conspecifics for suitable host in the choice assay may also lead to reduced number of eggs. Thus, in practice, it would be more beneficial for a farmer to grow more than one variety/line/species of amaranth in a mixed cropping system so as to reduce the pest burden or to interplant susceptible varieties, or avoid monocultures altogether.

Larval, pupal and total developmental time did not differ significantly among the tested accessions where development was successfully completed. This is probably due to similarity in the nutrient composition and quantities or composition of secondary compounds among the amaranth accessions [41]. Shorter developmental time of an insect pest on a host is usually an indicator of a more suitable host crop [41]. Jeyasankar and Gokilamani [4] reported mean larval, pupal and total development times of *S. recurvalis* to be 13 ± 3.0, 10 ± 2.0 and 25.5 ± 5.5 days, respectively on an amaranth variety, which is similar to our values of 13.5 ± 0.12, 6.36 ± 0.13 and 19.09 ± 0.15 days for larval, pupal and total developmental times, respectively. Similar developmental times of *S. recurvalis* were also recorded by Seham et al. [42] on sugar beet and Bhattacherjee and Ramdas-Menon [43] on an unknown plant species at 25 ± 2 °C. The slight variations in the developmental times of *S. recurvalis* may be due to differences in experimental conditions and host plants used or to differences in populations of *S. recurvalis*.

A high level of resistance was observed on accession VI036227 on which larval development of *S. recurvalis* could not proceed beyond the first instar. This could be a result of expression of antixenosis by the accession in which the plant produces feeding deterrents (volatiles) that prevent the larvae of *S. recurvalis* from feeding and resulting in death due to starvation. There is also a possibility of antibiosis where the plant possesses highly potent secondary metabolites that kill the pest larvae upon feeding on it. Secondary metabolites belonging to the group of phenolic acids were shown to have negative effects on insects by acting as deterrents or being toxic to non-adapted insects by inducing toxic oxidative stress on herbivores [7,44,45]. Hence, further studies are recommended to elucidate the bases of resistance of this accession in comparison with other resistant accessions with a special focus on the analysis of secondary metabolites and their role in pest resistance.

Larval mortality was highest on accession VI036227 (100%) and higher on accession VI056563 compared to RVI00053. High larval mortality on accession VI056563 is therefore an indication that it is unsuitable for the development of *S. recurvalis* in comparison to RVI00053. Negative *r_i_* values on all the accessions also indicate a decline in larval populations on these accessions. High larval mortality rates could be due to sub-optimal nutritional quality in the accession or presence of secondary metabolites that do not promote development of *S. recurvalis.* Early stage larval mortality was highest on accession VI036227 followed by VI056563 and was least on RVI00053. Voracious feeding by larvae of *S. recurvalis* usually begins after the second instar, when larvae can feed on entire foliage leaving only leaf veins intact [12,21,24]. Low mortality rates during the early stages of larval development would therefore result in greater damage inflicted on the plant as the larvae grow and feed. High early stage larval mortalities as observed on accession VI036227 is of critical importance and very desirable in the selection of resistant accessions because negligible damage is caused by larvae at this stage. Nevertheless, there was no significant difference in oviposition choice between this accession and the susceptible one, making it a ‘dead-end’ trap crop for *S. recurvalis*. On the contrary, accessions RVI00053, VI033479 and the susceptible check VI033482, which had low early stage mortalities, provide increased opportunities for the pest to cause extensive foliage damage as it matures. Pupal mortalities were higher on accessions RVI00053 and VI033479 compared to VI044437-A and were not correlated to larval mortalities suggesting that different compounds are responsible for mortality in the larvae and pupae of *S. recurvalis*. High larval and pupal mortalities have a significant role in reducing the populations of the pest in the subsequent generations and therefore accessions that lead to greater mortalities are highly desirable.

Apart from accession VI036227, which led to a significantly low weight gain when larvae of *S. recurvalis* were fed on it, weight gain from the other accessions did not differ significantly. The minimal weight gain on accession VI036227 (7.57% compared to >120% in other accessions) further reiterates the presence of either a feeding deterrent or a highly toxic secondary metabolite against larvae of *S. recurvalis*. Weight gain in the remaining accessions did not differ significantly, indicating that feeding by *S. recurvalis* larvae on the accessions was not deterred and suggests a lack of antixenosis for feeding in the accessions.

Significant differences in the longevity of adults of *S. recurvalis* raised on the different accessions were noted in our study, with accession VI047555-B producing adults with the shortest longevity. Shortened adult longevity is usually an indication of a less suitable host plant and is mainly attributed to low nutritional quality of that host plant [46]. Differences in the adult longevity of *S. recurvalis* were also reported between *Trianthema portulacastrum* L. (5.68 ± 0.7 days) and *Amaranthus* sp. (4.99 ± 0.3 days) [47] in Taiwan. Pande [48] also reported short adult longevities of between 3.5 and 6 days in males and females, respectively on *Trianthema monogyna* L. In contrast, Shirai [49] reported extended adult longevities of 18.8±7.6 days and 15.1 ± 6.9 days in females and males of *S. recurvalis,* respectively when fed on spinach leaves (*Spinacia oleracea* L.) and Seham et al. [42] reported longevities of 28.46 ± 1.88 and 26.08 ± 1.83 in females and males of *S. recurvalis*, respectively, when fed on sugar beet (*Beta vulgaris* L.). Although this broad variation in *S. recurvalis* adult longevities can be due to the differences in experimental conditions as in Seham et al. [42] at 18.6 ± 2 °C and 70 ± 5% RH, the host plant on which the pest develops could play a big role [47]. Other studies involving lepidopteran pests when reared on different host plants including *Helicoverpa armigera* Hübner [46] have also shown differences in adult longevity. Thus, shortened adult longevity can be attributed to expression of antibiosis by the host plant or inadequate nutrition in the host plant.

The accession VI036227 exhibited exemplary antibiotic traits by causing 100% pest mortality. However, it possessed very undesirable morphological/agronomic traits mainly tiny leaves (more than 6 times smaller than susceptible check), slow germination and prostate growth habit. In East Africa, vegetable leaf yield is of high importance to both farmers and breeders [3], posing the challenge of acceptability to this resistant accession by farmers and consumers. The other seven accessions had traits such as moderate mortality rates on accession VI056563 and elicited lower rates of oviposition compared to the susceptible check. They also possess better morphological/agronomic traits compared to VI036227, including erect growth habit and large/broad leaves, which may result in high vegetable leaf yields and might easily be accepted by local farmers and breeders. Whether the desirable antibiotic trait of accession VI036227 can be transferred to confer resistance to locally cultivated varieties and other accessions of amaranth is still unclear and is strongly recommended for future studies. Further studies are also recommended to assess farmers’ and consumers’ preferences and acceptance/willingness to cultivate and consume these pest resistant accessions. The yield potential, storability, drought tolerance and nutritive attributes of these different resistant varieties under various agro-ecological conditions also warrant further research. 

## 5. Conclusions

The assessed amaranth accessions expressed both antixenotic and antibiotic resistance traits against *S. recurvalis*. Antixenosis traits exhibited through non-preference for oviposition were highly expressed in several accessions including VI044432, VI049502, VI054569 and VI048076. Larval development was completely hindered on accession VI036227, resulting in 100% larval mortality and points to presence of potent antibiosis. In addition, VI036227 showed no antixenosis, suggesting further potential as a ‘dead-end’ trap crop. Early stage larval mortality, total larval and pupal mortalities as well as adult longevity were moderate on accessions VI048076, VI056563 and VI047555-B suggesting moderate level of antibiosis. HPR to insect pests forms the core of many IPM programs [50,51] but is seldom exploited for pest management among TLVs. This is despite the fact that HPR is not only compatible with environmental concerns and other pest management strategies, but also significantly reduces pest control expenses [46], since the pest management solution is inherent in the crop. The accessions expressing adverse effects on the biology of *S. recurvalis* are thus recommended for evaluation for an IPM package for the management of the pest.

## Figures and Tables

**Table 1:** Amaranth accessions, lines developed by single plant selection from germplasm collections studied and some of their morphological characteristics.

Gene Bank Code	Species	Type	Leaf Colour	Leaf Shape	Country of Origin	Number of Branches per Plant (mean)	Plant Height (mean) cm	Leaf Width (mean) cm	Leaf Length (mean) cm	Petiole Length (mean) cm
VI033482 *	*A. tricolor* L.	Accession	Green	Reniform	Malaysia	9.0	100.9	10.6	19.3	5.0
RVI00002	*A. cruentus* L.	Line	Green	Ovate	Zambia	12.5	122.6	6.6	16.5	10.2
RVI00005	*A. dubius* Mart.	Line	Light reddish	Ovate	Tanzania	12.2	140.3	6.0	12.5	7.6
RVI00027	*Amaranthus* sp. 1	Line	Green	Ovate	Malawi	7.3	96.3	6.2	11.1	7.8
RVI00053	*A. dubius* Mart.	Line	Green	Ovate	Uganda	11.0	167.0	8.7	15.6	8.0
VI033477	*Amaranthus* sp. 2	Accession	Reddish	Ovate	Malaysia	9.2	99.7	7.9	12.3	5.2
VI033479	*Amaranthus* sp. 3	Accession	Green	Ovate	Malaysia	11.3	100.8	4.9	7.6	4.2
VI033487	*A. cruentus* L.	Accession	Green	Reniform	Malaysia	13.5	128.7	5.6	7.7	6.6
VI036225	*A. graecizans* L.	Accession	Green	Ovate	Hungary	15.4	77.2	1.6	3.2	2.3
VI036227	*A. blitoides* Watson	Accession	Green	Oblanceolate	Hungary	15.8	67.4	1.2	3.2	1.6
VI044367	*A. cruentus* L.	Accession	Green	Lanceolate	Tanzania	9.1	123.5	5.8	13.7	10.2
VI044369	*A. hypochondriacus* L.	Accession	Green	Lanceolate	Ghana	13.7	129.0	6.1	17.0	9.2
VI044388	*A. graecizans* L.	Accession	Green	Oblanceolate	India	14.6	89.8	2.3	4.3	2.5
VI044432	*A. viridis* L.	Accession	Green	Cordate	Indonesia	11.0	102.9	4.5	6.9	4.0
VI044437-A	*A. cruentus* L.	Accession	Green	Lanceolate	Malaysia	11.5	89.5	5.5	13.0	7.2
VI044473	*A. palmeri* Watson	Accession	Green	Obovate	Senegal	9.0	80.1	2.2	4.4	2.4
VI046233-A	*Amaranthus* sp. 4	Accession	Reddish	Lanceolate	Vietnam	8.0	142.1	6.8	17.0	10.6
VI047517-B	*A. tricolor* L.	Accession	Green	Ovate	Bangladesh	12.9	119.8	8.1	15.9	7.7
VI047555-B	*A. tricolor* L.	Accession	Green	Lanceolate	Vietnam	10.9	135.6	4.5	13.4	4.8
VI048076	*A. tricolor* L.	Accession	Green	Cordate	Bangladesh	13.1	130.1	8.0	13.5	7.1
VI048864-A	*A. viridis* L.	Accession	Green	Cordate	Thailand	10.2	95.1	4.1	5.8	3.6
VI048919	*Amaranthus* sp. 5	Accession	Green	Ovate	Thailand	11.6	126.1	3.7	7.0	4.3
VI049242	*Amaranthus* sp. 6	Accession	Green	Ovate	Thailand	11.6	87.8	4.4	5.8	3.7
VI049502	*Amaranthus* sp. 7	Accession	Green	Cordate	Thailand	10.0	103.1	4.7	6.8	4.1
VI049504	*Amaranthus* sp. 8	Accession	Green	Lanceolate	Thailand	12.1	134.2	3.0	6.6	3.2
VI049530	*Amaranthus* sp. 9	Accession	Green	Ovate	Thailand	10.4	89.2	4.3	6.4	3.9
VI049639	*A. viridis* L.	Accession	Green	Ovate	Thailand	11.3	91.7	4.2	6.1	3.5
VI049698	*A. viridis* L.	Accession	Green	Ovate	Thailand	12.4	100.5	3.9	5.5	3.4
VI050609-A	*A. tricolor* L.	Accession	Variegated	Cordate	Vietnam	9.8	129.1	9.2	11.7	6.8
VI050609-B	*A. tricolor* L	Accession	Variegated	Ovate	Vietnam	8.5	140.9	9.6	15.0	6.1
VI054569	*A. gracilis* Desf.	Accession	Green	Ovate	Philippines	11.0	95.1	4.5	7.2	3.9
VI054798	*Amaranthus* sp. 10	Accession	Green	Ovate	Lao PDR	12.4	89.3	4.1	6.2	3.3
VI055127	*A. viridis* L.	Accession	Green	Ovate	Malaysia	11.4	108.1	5.6	10.3	6.6
VI055128	*A. viridis* L.	Accession	Green	Cordate	Malaysia	10.7	123.4	5.0	7.0	3.9
VI055135	*A. viridis* L	Accession	Green	Cordate	Malaysia	10.8	92.0	5.0	7.2	4.1
VI056563	*Amaranthus* sp. 11	Accession	Reddish	Ovate	Bangladesh	9.7	136.9	9.0	17.1	8.3
Mean						11.2	110.7	5.5	10.0	5.5
*p*-value						0.053	<0.001	<0.001	<0.001	<0.001
LSD (5%)						4.38	26.74	1.4	3.69	2.33

* Susceptible check.

**Table 2:** Number of eggs (mean ± SE) laid by *S. recurvalis* on different accessions of amaranth in the choice assays in set-up A and B.

Set-up A			Set-up B		
Gene Bank Code	Number of Eggs	Relative Risk	Gene Bank Code	Number of Eggs	Relative Risk
VI033482 *	25.67 ± 7.62a		VI033482*	29.00 ± 7.95b	
VI033487	11.33 ± 4.57b	0.44	VI048919	40.20 ± 16.41a	1.39
VI044388	10.00 ± 2.92bc	0.39	VI050609-B	34.67 ± 9.47ab	1.2
VI036227	8.83 ± 3.93bcd	0.34	VI033477	15.00 ± 4.87c	0.52
RVI00027	7.33 ± 2.85be	0.29	VI049504	11.40 ± 6.21cd	0.39
RVI00005	6.17 ± 2.30cf	0.24	VI047517-B	9.83 ± 4.48cde	0.34
VI048076	6.17 ± 1.64cf	0.24	VI056563	9.83 ± 3.51cde	0.34
VI049639	4.33 ± 1.74def	0.17	VI055127	6.00 ± 3.04def	0.21
RVI00002	4.00 ± 2.08ef	0.16	VI046233-A	6.17 ± 3.82def	0.21
RVI00053	3.83 ± 1.70ef	0.15	VI049530	6.67 ± 3.48def	0.23
VI036225	3.67 ± 2.01ef	0.14	VI050609-A	6.67 ± 2.89def	0.23
VI044473	3.33 ± 1.54ef	0.13	VI047555-B	3.67 ± 1.87ef	0.13
VI044369	3.20 ± 2.03ef	0.12	VI055128	3.50 ± 2.05ef	0.12
VI044367	2.83 ± 0.83ef	0.11	VI054798	2.83 ± 2.46f	0.1
VI044437-A	2.40 ± 1.03ef	0.09	VI055135	2.50 ± 0.92f	0.09
VI049698	2.33 ± 0.56f	0.09	VI048864-A	2.33 ± 1.76f	0.08
VI044432	1.50 ± 0.56f	0.06	VI033479	2.17 ± 1.78f	0.07
VI054569	1.50 ± 0.85f	0.06	VI049242	2.17 ± 1.17f	0.07
			VI049502	1.50 ± 0.81f	0.05

* Susceptible accession. Means followed by same lower-case letter within a column are not significantly different at *p* < 0.05 (Tukey’s test).

**Table 3:** Number of eggs (mean ± SE) laid on selected accessions in the no-choice and choice assays.

Gene Bank Code	No Choice	Relative Risk	Choice	Relative Risk	χ^2^	df	*p* Value
VI033482 *	59.63 ± 10.49eA	-	21.58 ± 5.30dB	-	127.96	1	<0.001
RVI00053	30.00 ± 6.52bcA	0.50	3.83 ± 1.70abB	0.18	183.54	1	<0.001
VI036227	40.20 ± 10.24cdA	0.67	8.83 ± 3.93cB	0.41	170.15	1	<0.001
VI044437-A	29.33 ± 10.24bA	0.49	2.40 ± 1.03abB	0.11	282.65	1	<0.001
VI047555-B	29.40 ± 10.27bcA	0.49	3.67 ± 1.87abB	0.17	181.96	1	<0.001
VI048076	18.50 ± 6.63aA	0.31	6.17 ± 1.64bcB	0.29	72.01	1	<0.001
VI049698	42.80 ± 18.14dA	0.72	2.33 ± 0.56aB	0.11	347.38	1	<0.001
VI056563	37.20 ± 5.54bcdA	0.62	9.83 ± 3.51cB	0.46	129.6	1	<0.001
	χ^2^ =192.75		χ^2^ =281.29				
	df = 7, 37		df = 7, 45				
	*p* < 0.001		*p* < 0.001				

* Susceptible accession. Means followed by the upper-case letters within rows or lower-case letter within columns are not significantly different at *p* < 0.05 using Tukey’s test (chi-square test).

**Table 4:** Larval, pupal and total developmental times (mean ± SE) (days) of *S. recurvalis* on selected amaranth accessions and early stage (36 h) larval mortality on each accession.

Gene Bank Code	Larval Development Time	Pupal Development Time	Total Development Time	Early Stage Larval Mortality (%)
VI033482 *	13.43 ± 0.30a	7.45 ± 0.69a	20.00 ± 0.8a	8.0 ± 3.27bc
RVI00053	13.45 ± 0.37a	6.60 ± 0.34a	18.60 ± 0.45a	4.0 ± 0.51c
VI033479	13.24 ± 0.23a	6.11 ± 0.35a	18.67 ± 0.44a	8.0 ± 3.27bc
VI036227	NA	NA	NA	100.0 ± 0.0a
VI044437-A	13.29 ± 0.29a	5.86 ± 0.17a	18.82 ± 0.28a	14.0 ± 5.21bc
VI047555-B	13.77 ± 0.36a	6.16 ± 0.21a	19.11 ± 0.24a	24.0 ± 6.53bc
VI048076	13.77 ± 0.43a	6.20 ± 0.43a	19.00 ± 0.45a	22.0 ± 7.57bc
VI049698	13.23 ± 0.30a	6.43 ± 0.39a	19.24 ± 0.36a	12.0 ± 6.11bc
VI056563	14.00 ± 0.56a	6.63 ± 0.42a	19.75 ± 0.53a	34.0 ± 11.57b
	χ^2^ = 1.066	χ^2^ = 3.348	χ^2^ = 1.042	*F* = 12.22
	df = 7, 228	df = 7, 112	df = 7, 112	df = 8, 81
	*p* = 0.994	*p* = 0.851	*p* = 0.994	*p* < 0.001

* Susceptible accession. Means followed by same lower-case letter within a column are not significantly different at *p* < 0.05 (Tukey’s test).

**Table 5:** *Spoladea recurvalis* adult longevity, egg viability (%), fecundity and proportion of F_1_ females (mean ± SE) on selected amaranth accessions.

Gene Bank Code	Adult Longevity (Days)	Odds Ratio	Egg Viability (%)	Fecundity/Female at 4-d-old	Proportion of Females (%)
VI047555-B	8.70 ± 0.61a	0.9	91.25 ± 1.93a	13.06 ± 1.20ab	51.79 ± 7.03a
VI033482 *	9.69 ± 0.85ab		95.49 ± 1.50a	22.67 ± 3.67a	59.17 ± 8.86a
VI044437-A	10.85 ± 0.52b	1.12	97.79 ± 3.13a	11.09 ± 0.80b	44.97 ± 8.01a
VI033479	10.85 ± 0.85b	1.12	97.62 ± 1.61a	10.97 ± 1.71b	45.49 ± 4.51a
RVI00053	11.00 ± 0.79bc	1.14	97.53 ± 1.51a	13.40 ± 1.74ab	66.91 ± 8.09a
VI049698	11.22 ± 0.49bc	1.16	96.25 ± 2.33a	23.75 ± 3.50a	46.03 ± 6.31a
VI056563	12.63 ± 0.82cd	1.3	95.90 ± 4.10a	14.67 ± 5.67ab	49.77 ± 5.83a
VI048076	14.25 ± 0.82d	1.47	94.66 ± 2.59a	8.50 ± 0.74b	56.76 ± 13.44a
	*p* < 0.001		*p* = 0.527	*p* = 0.002	*p* = 0.638
	df = 7, 380		df = 7, 32	df = 7,14	df = 7,25
	χ^2^ = 92.51		*F* = 0.89	*F* = 6.07	*F* = 0.74

* Susceptible accession. Means followed by the same lower-case letter within a column are not significantly different at *p* < 0.05 (Tukey’s test).
